# Inversion of chromosome 7q22 and q36 as a sole abnormality presenting in myelodysplastic syndrome: a case report

**DOI:** 10.1186/1752-1947-8-268

**Published:** 2014-08-05

**Authors:** Hiroto Kaneko, Kazuho Shimura, Saeko Kuwahara, Muneo Ohshiro, Yasuhiko Tsutsumi, Toshiki Iwai, Shigeo Horiike, Shouhei Yokota, Yasuo Ohkawara, Masafumi Taniwaki

**Affiliations:** 1 Department of Hematology, Japanese Red Cross Kyoto Daiichi Hospital, 15-749 Honmachi, Higashiyama-ward, Kyoto 605-0981, Japan; 2 Department of Hematology, Aiseikai-Yamashina Hospital, 19-4 Takehana-Shichouno-cho, Yamashina-ward, Kyoto 607-8086, Japan; 3 Department of Hematology/Oncology, Kyoto Prefectural University of Medicine, 465 Kajii-cho, Kawaramachi-Hirokoji, Kamigyo-ward, Kyoto 602-8566, Japan

**Keywords:** Chromosome 7q22, Inversion, Myelodysplastic syndrome

## Abstract

**Introduction:**

Deletions of chromosome 7 are often detected in myelodysplastic syndrome. The most commonly deleted segments are clustered at band 7q22. A critical gene is therefore suggested to be located in this region. We report a patient with myelodysplastic syndrome whose marrow cells carried an inversion of 7q22 and q36 as a sole karyotypic abnormality. How this extremely rare chromosomal aberration contributes to the pathogenesis of myelodysplastic syndrome should be clarified by accumulating clinical data of such cases.

**Case presentation:**

A 74-year-old Japanese man presented with pancytopenia incidentally detected by routine medical check-up. His complete blood cell counts revealed that his white blood cells had decreased to 2100/mm^3^, neutrophils 940/mm^3^, red blood cells 320×10^4^/mm^3^, hemoglobin 11.1g/dL, hematocrit 33.1%, and platelets 12.6×10^4^/mm^3^. Bone marrow examination showed normal cellularity with nucleated cells of 9.4×10^4^/mm^3^. The proportion of blasts was 4%. A morphological examination showed only basophilic stippling of erythroblasts which was seen as dysplasia. According to World Health Organization classification, the diagnosis was myelodysplastic syndrome-u. Karyotypic analysis showed 46,XY,inv(7)(q22q36) in all of 20 metaphases examined. Additional analysis revealed the karyotype of his lymphocytes was 46,XY. He is asymptomatic and cytopenia has slowly progressed.

**Conclusions:**

To the best of our knowledge, this karyotype from a clinical sample of *de novo* malignancies has never been documented although the identical karyotype from secondary myelodysplastic syndrome was reported. Despite the extremely low frequency, inversion of 7q22 appears to play a crucial role for myelodysplastic syndrome in this patient.

## Introduction

Chromosomal abnormalities are found in a considerable number of patients with myelodysplastic syndrome (MDS). Partial or whole deletions of the long arm of chromosome 7 (7q) are often detected [1]. Abnormality of chromosome 7q, especially as a part of complex chromosomal abnormalities, is clearly associated with poor prognosis [2]. Previous studies in myeloid malignancies have pinpointed the most commonly deleted segments in chromosome band 7q22 [3-6]. The importance of chromosomal rearrangement is more enhanced when it is detected as a sole aberration. We report a patient with MDS whose marrow cells carried only an inversion of 7q22 and q36.

## Case presentation

**Figure 1:**
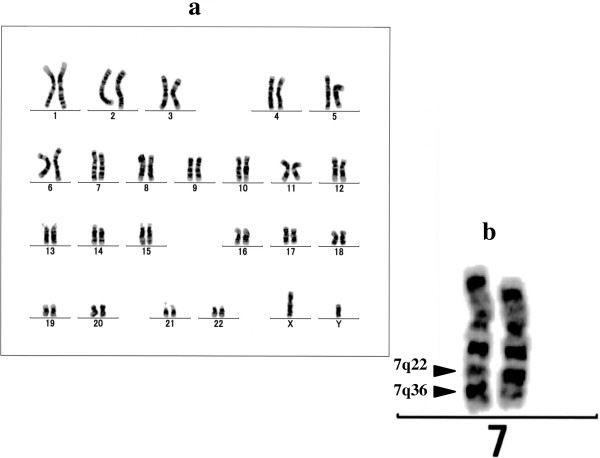
**Karyotype of marrow cells. a**. 46,XY,inv(7)(q22q36) was seen in all of 20 examined metaphases. **b**. Enlarged chromosomes 7 are also shown.

A 74-year-old Japanese man presented with pancytopenia incidentally detected by routine medical check-up. His complete blood cell counts revealed white blood cells decreased to 2100/mm^3^, neutrophils 940/mm^3^, red blood cells 320×10^4^/mm^3^, hemoglobin 11.1g/dL, hematocrit 33.1%, and platelets 12.6×10^4^/mm^3^. His serum lactate dehydrogenase remained within the normal limit at 148IU/L. A bone marrow examination showed normal cellularity with nucleated cells of 9.4×10^4^/mm^3^. The proportion of blasts was 4%. A morphological examination showed basophilic stippling of erythroblasts which was seen as dysplasia. No pseudo-Pelger anomaly or hypogranular neutrophils, megaloblastic change of erythroblasts, or micromegakaryocytes or giant platelets were detected. According to World Health Organization classification [7], the diagnosis was MDS-u. Using our previously established method [8], the karyotype of his marrow cells were analyzed [9]; 46,XY, inv(7)(q22q36) in all of 20 metaphases was shown (Figure 1). Additional chromosomal analysis of his blood lymphocytes stimulated by phytohemagglutinin-A was 46, XY. This indicated that inv(7)(q22q36) seen in his bone marrow cells was not a constitutional aberration. Unfortunately, further molecular examination was not carried out. Although he was assigned a score of intermediate-2 using the International Prognostic Scoring System [2], he is now asymptomatic and cytopenia has progressed slowly for 4 years.

## Discussion

To the best of our knowledge, inv(7)(q22q36) as a sole chromosomal aberration in a clinical sample of *de novo* malignancies has never been documented. Among 5345 clinical samples from various neoplasms reviewed by Mitelman, the identical karyotype was reported once from a case of secondary MDS [10]. Knuutila *et al*. described that radiation therapy for Hodgkin’s lymphoma had been given before MDS in this case [11]. Todd *et al*. described an inversion involving 7q22 and q34 as a sole abnormality in cell line established from familial MDS [12]. Taken together, despite the extremely low frequency, this karyotype appears to play a certain role in MDS pathogenesis.

Chromosome band 7q22 is involved in as many as 80% of 7q deletions seen in MDS and acute leukemia [3]. Thus, it is suggested that a critical gene for MDS development might be located at 7q22. Recent observation that *CUX1* (cut-like homeobox 1, encoding a transcription factor located at chromosome 7q22), is frequently deleted in myeloid neoplasms is noteworthy [6]. Inactivating mutation is reported to lead tumorigenesis in various types of malignancies [13]. Loss of *CUX1* function in myeloid malignancies is additionally suggested to be associated with poor prognosis [14]. Although our patient showed no leukemic transformation or rapid progression of cytopenia and is uneventfully alive, indicating a favorable prognosis, this gene might be disrupted since the breakpoint of 7q inversion of our patient also involves chromosome band 7q22. Whether this gene is rearranged in inv(7)(q22q36) should be examined to evaluate pathogenic significance. Taken together, despite the extremely low frequency, this karyotype appears to play a certain role in MDS pathogenesis.

## Conclusion

Inv(7)(q22q36) appears to play a crucial role in MDS in a small proportion of patients.

## Consent

Written informed consent was obtained from the patient for publication of this case report and any accompanying images. A copy of the written consent is available for review by the Editor-in-Chief of this journal.

## Abbreviations

MDS: Myelodysplastic syndrome.

## Competing interests

The authors declare that they have no competing interests.

## Authors’ contributions

HK examined and diagnosed the patient’s disease and treated the patient. HK also wrote draft manuscript. KS helped in the analysis of the bone marrow data and refined the manuscript. SH, SY and MT analyzed the karyotype of the patient and researched previously reported similar cases. SK, MO, YT, and TI assisted in the therapeutic management of the patient. YO critically read the manuscript. All authors read and approved the final manuscript.
